# Learning algorithms for oscillatory neural networks as associative memory for pattern recognition

**DOI:** 10.3389/fnins.2023.1257611

**Published:** 2023-11-29

**Authors:** Manuel Jiménez, María J. Avedillo, Bernabé Linares-Barranco, Juan Núñez

**Affiliations:** Instituto de Microelectrónica de Sevilla, IMSE-CNM (CSIC/Universidad de Sevilla), Seville, Spain

**Keywords:** oscillatory neural networks (ONNs), associative memory, pattern recognition, character recognition, machine learning algorithms, hopfield neural networks, oscillators, phase-change material

## Abstract

Alternative paradigms to the von Neumann computing scheme are currently arousing huge interest. Oscillatory neural networks (ONNs) using emerging phase-change materials like VO_2_ constitute an energy-efficient, massively parallel, brain-inspired, in-memory computing approach. The encoding of information in the phase pattern of frequency-locked, weakly coupled oscillators makes it possible to exploit their rich non-linear dynamics and their synchronization phenomena for computing. A single fully connected ONN layer can implement an auto-associative memory comparable to that of a Hopfield network, hence Hebbian learning rule is the most widely adopted method for configuring ONNs for such applications, despite its well-known limitations. An extensive amount of literature is available about learning in Hopfield networks, with information regarding many different learning algorithms that perform better than the Hebbian rule. However, not all of these algorithms are useful for ONN training due to the constraints imposed by their physical implementation. This paper evaluates different learning methods with respect to their suitability for ONNs. It proposes a new approach, which is compared against previous works. The proposed method has been shown to produce competitive results in terms of pattern recognition accuracy with reduced precision in synaptic weights, and to be suitable for online learning.

## 1 Introduction

Today’s computing platforms face the challenge of having to handle a large amount of information and complex operations. This has an impact on their power consumption, in addition to the challenge of the so-called bottleneck in Von Neumann-type architectures. On the other hand, conventional CMOS technology is physically limited by its energy efficiency, as its continuous scaling results in higher losses due to leakage currents. There is therefore growing interest in alternative computing paradigms such as in-memory computing or oscillatory-based computing (OBC) (Hoppensteadt and Izhikevich, 2000; Csaba and Porod, 2020), capable of combining highly parallel information processing with the attraction of energy-efficient operation. OBC exploits the rich, non-linear, oscillatory dynamics to implement mathematical functions that link up a system’s output and input states.

Within OBC, there are different approaches depending on how the oscillators interact. For example, they can have identical frequency and encode information in their phase differences (phase-shift keying, PSK) or they can work at different operating frequencies (frequency-shift keying). Weakly coupled oscillators and their synchronization phenomena stands for a promising way of implementing OBC.

These coupled oscillators are implemented with novel emerging devices that are potentially attractive due to their low area requirement and their high capacity to operate with low power consumption, as the result of their particular electrical operating mechanisms. VO_2_ devices, in particular, stand out for their hysteresis in the characteristic I-V curve, which makes it possible to easily implement relaxation oscillators (Núñez et al., 2021).

**FIGURE 1:**
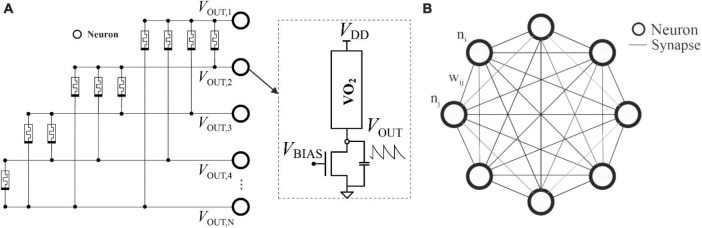
**(A)** VO_2_-based ONN, coupled with memristors. Each circle represents the nano-oscillator/neuron circuit shown in the dotted view to the right. The states are determined by the difference between their phases; **(B)** HNN topology, where each edge connection is assigned to the synaptic weight (*w*_*ij*_) between two neurons (*n_i_* and *n_j_*), marked as circles. Each neuron has a state value associated with its activation function, Eq. (1).

The connection of a multitude of oscillator circuits by means of electrical elements which act as synapses creates an intelligent collective system called an oscillatory neural network (ONN). PSK-based ONN encodes information in the relationship between oscillator phases. The input presented to the ONN is applied controlling the initial phase differences of the oscillators through circuit polarization. Figure 1A shows an analog ONN design using VO_2_-based nano-oscillators as neurons and resistively weak couplings as synapses. ONNs mimic the functioning of certain neurons in particular areas of the brain which, thanks to their synchrony, are able to process and transmit information quickly and efficiently. This processing by synchronism is not exclusive to the human body and can be found elsewhere in nature: for example, in the central pattern generators and synchronized locomotion of animals (Dutta et al., 2019), in the flashing rhythms of fireflies, or in the interaction between coupled pendulum clocks.

Structurally, the ONN illustrated resembles an artificial network called the Hopfield Neural Network (HNN) (Hopfield, 1982), which has been studied in depth with regard to associative memory (AM) and pattern recognition tasks. As can be seen in Figure 1B, the HNN has a simple conceptual model comprising a single, recurrent, fully connected layer of neurons with synaptic weights trained to keep given network states as stable fixed points.

The HNN mathematical model can therefore be useful when studying the behavior of the ONN and obtaining results prior to electrical simulations or experimental evaluation. To translate trained weights into feasible bidirectional ONN couplings, however, the physical constraints of electrical synapses must be considered (Hopfield, 1982; Corti et al., 2018; Corti et al., 2020; Núñez et al., 2021; Shamsi et al., 2021). Suitable training solutions and mathematical model parameters are limited by certain essential design aspects, such as how many different coupling strengths can be discerned, and it is therefore considered essential to study the precision of discrete weights using the mathematical model.

The rest of the paper is organized as follows. Section “2. Materials and methods” starts with a description of the Hopfield model and highlights some important considerations regarding the training process. It also presents the most important learning rules for AM operation and introduces the iterative learning algorithm developed by the authors. This Section describes, as well, the pattern recognition experiment carried out as benchmark. Section “3. Results” presents the results obtained for capacity and noise robustness using our approach. The approach is also compared with other well-known learning methods. Finally, section “4. Discussion” summarizes the conclusions.

## 2 Materials and methods

### 2.1 Hopfield neural network and learning rules

#### 2.1.1 Hopfield model

The HNN topology is a special kind of recurrent neural network in which all *N* neurons are interconnected with each other in a single layer with given weights. *w*_*ij*_ denotes the weight associated with the connection between neurons *i* and *j*. In the discrete, synchronous version of the HNN, each neuron state, *s_i_*, operates with bipolar values {−1, + 1} and is simultaneously updated as:


(1) $$s_{i}=s\,i\,g\,n\,\left(\sum_{j=1}^{N}w_{i\,j}\,s_{j}\right)=s\,i\,g\,n\,\left(h_{i}\right)$$

The neuron’s hidden potential, *h*_*i*_, is defined as the internal value within the activation *sign* function in Eq. (1). In this way, the network behavior gradually transitions from an initial input state to one of the previously stored states, determined by the synaptic weights. After network training, the weight values are assigned in such a way that the patterns to be stored are fixed as attractor states, associated with local minimum energy points of the Hamiltonian energy function:


(2) $$E=-\frac{1}{2}\,\sum_{i=1}^{N}\sum_{j=1}^{N}w_{i\,j}\,s_{i}\,s_{j}=-\frac{1}{2}\,\sum_{i=1}^{N}h_{i}\,s_{i}$$

Note that the transcribed equations deliberately omit the use of bias parameters for the reasons discussed below.

The AM operation thus distinguishes between two different steps: training (a.k.a. learning) and inference.

#### 2.2.2 Learning considerations in ONNs

Learning is the process of obtaining appropriate values for weights, thereby compressing the information to be stored in the relationships between the weight values. The weights, which can be grouped into a matrix, determine the synaptic strengths—and therefore determine the interactions—between neurons.

Given that ONN synapses comprise electrical couplings, the computed weights cannot be directly translated to electrical conductance parameters: a mapping step is required (Delacour and Todri-Sanial, 2021). Furthermore, physical implementation imposes restrictions on the weight matrix: bidirectional coupling in oscillator connections requires the matrix to be symmetric; weight precision is limited by how many electrical strengths the system can distinguish; and layout parasitics can impact the performance by modifying the effective synaptic strength and operating frequency. The original HNN also includes a bias parameter representing the continuous contribution of one neuron with a fixed state. Since the ONN architecture does not include such a contribution, this parameter is not included in the model.

#### 2.2.3 Training of associative memory neural networks

Training algorithms found in Machine Learning are typically classified as supervised or unsupervised, on the basis of whether or not knowledge of the target output is used during learning. Depending on the conditions in which the learning is executed, distinctions can also be made between one-shot or iterative, incremental or non-incremental, and local or non-local learning. A learning method is incremental when it only uses information from the new pattern to learn and the current weight matrix to compute the new weight matrix, whereas it is iterative if it makes several presentations of the same information to the training algorithm until it is learned. Locality occurs if the increment in the weight between two neurons only depends on the desired state for those two neurons, or its hidden potential. This is an attractive property because it makes learning biologically plausible.

Depending on its implementation, learning can also be considered offline or online on the basis of whether it needs to know the network’s response before updating the weights.

Different learning algorithms have been reported in the past to successfully address the problem of training HNNs to retain different steady states.

The most widespread is the well-known Hebbian learning rule: if neurons are activated together, the strength of their mutual coupling will increase, while otherwise the connection will be weaker (Hebb, 1949).

Thus, for a set of target *P* patterns of length *N*, *ξ^k^* ∈ {−1, + 1}^*N*^, to be stored, the coupling strength between neurons corresponds to the correlation of their state values and can be determined as:


(3) $$w_{i\,j}=\sum_{k=1}^{P}\xi_{i}^{k}\,\xi_{j}^{k}$$

where component $\xi_{i}^{k}$ represents the desired state of neuron *i* when pattern *k* shall be retrieved.

Memory capacity limitations (Folli et al., 2017) storing highly correlated patterns stimulated further research into learning mechanisms. Storkey (1997) proposed an alternative rule that considers current knowledge of the network during the training steps. That is to say, the weight updates are obtained considering the previous weights.


(4) $$w_{i\,j}^{k}=w_{i\,j}^{k-1}+\frac{\xi_{i}^{k}\,\xi_{j}^{k}}{N-1}\,\left(\xi_{i}^{k}\,h_{j\,i}^{k}+h_{i\,j}^{k}\,\xi_{j}^{k}\right)\,h_{i\,j}^{k}=\sum_{l=1,l\neq i,j}^{N}w_{i\,l}^{k-1}\,\xi_{l}^{k}$$

This algorithm serves to remove lower-order noise associated with the interaction of the different attractors, where subtraction is applied by computing pre-synaptic and post-synaptic forms of the local field: $h_{ji}^{k}$ and $h_{ij}^{k}$. It has been demonstrated (Storkey, 1997; Storkey and Valabregue, 1997; Folli et al., 2017), that while the absolute capacity of the Hebbian algorithm (no retrieval errors allowed) is given by ${\text{C}}_{\text{abs}}=\frac{N}{2\,\ln\left(N\right)}$, Storkey’s rule achieves ${\text{C}}_{\text{abs}}=\frac{N}{\sqrt{2\,\ln\left(N\right)}}$.

Both learning rules feature a one-shot, local, incremental, unsupervised training method. However, a most recent work (Jiménez-Través et al., 2022) shows that Storkey’s learning rule can be improved as AM by adopting a few-shot approach and training the network with more than one repetition of the training set to be stored.

Other authors (Diederich and Opper, 1987) have analyzed pattern embedding conditions during training to solve the problem of weight assignment. A set of patterns constitutes fixed points in the neural network if the following set of conditions is satisfied:


(5) $$\sum_{j=1}^{N}w_{i\,j}\,\xi_{j}^{k}\,\xi_{i}^{k}=h_{i}^{k}\,\xi_{i}^{k}>0\,\begin{array}{c} \forall i\in 1,\ldots ,N\\ \forall k\in 1,\ldots ,P \end{array}$$

Thus, weights (*w*_*ij*_) are obtained by solving this system of *N⋅P* constraints. Note that the pattern-embedding conditions are satisfied when each neuron potential ($h_{i}^{k}$) is aligned with the target state value, $\xi_{i}^{k}$, for each pattern. Alignment requires that both have the same sign. Derivation of equation (5) is described in Supplementary material.

In this training algorithm, a threshold value *T* can also be applied substituting 0 in (5). It is employed to increase the basins of attraction associated to the stored patterns, which are fixed points of the network. If *T* = 1, for example, the solution to (5) is the orthogonal projection matrix of the network’s state space over the set of training patterns (Personnaz et al., 1986). This matrix has good properties to function as an associative memory, even with correlated patterns (Personnaz et al., 1986).

Personnaz et al. (1985) developed a direct analytical method to obtain the orthogonal projection matrix. However, this method was computationally intense and posed serious challenges for hardware implementation.

A wide variety of solutions to the weight assignment problem also resort to optimization solvers (Tolmachev and Manton, 2020). Algorithms using this approach define an objective function to minimize, such as different distance metrics, and iteratively solve the optimization problem with the gradient descent error. Again, however, these solutions have similar disadvantages regarding hardware implementation.

Two simple iterative methods for solving the pattern-embedding problem were presented in Diederich and Opper (1987). The first is called D&O’s Rule I (flow diagram of this rule is shown in Supplementary material). Here, for each pattern *k*, neuron responses are checked sequentially one by one to assess whether the current weights require updating following a failure to satisfy the alignment condition (5) with a threshold value *T*. A larger *T-*value implies more training iterations, as long as higher weights are required to accomplish condition (5). Rule I algorithm performs the weights update applying Hebb’s rule scaled by (*N*-1) and focused on *T* = 1. This process is repeated until the algorithm converges to a solution that satisfies condition (5) for all *NP* constraints. This solution is mathematically demonstrated in Diederich and Opper (1987) for an asymmetric weight matrix since it does not couple different rows, that is, weights are not bidirectional. This learning rule can be summarized using the Heaviside function, θ, as follows:


(6) $$△\,w_{i\,j}=\frac{\xi_{j}^{k}\,\xi_{i}^{k}}{N-1}\,\theta \,\left(T-h_{i}^{k}\,\xi_{i}^{k}\right)\,\begin{array}{c} \forall i\in 1,\ldots ,N\\ \forall k\in 1,\ldots ,P \end{array}$$

The second rule, D&O’s Rule II, implements a similar process, but the weights are incremented as:


(7) $$△\,w_{i\,j}=\frac{\xi_{j}^{k}\,\xi_{i}^{k}}{N-1}\,\left(T-h_{i}^{k}\,\xi_{i}^{k}\right)\,\begin{array}{c} \forall i\in 1,\ldots ,N\\ \forall k\in 1,\ldots ,P \end{array}$$

This is the distinct of what happens in Rule I, where constant increments/decrements are applied. In Rule II, the weight increment is also proportional to Hebb’s update rule scaled by (*N*-1). When the product of the neuron potential, $h_{i}^{k}$, and the target value to store, $\xi_{i}^{k}$, approaches *T* (coinciding in sign), the weight increment is progressively reduced to zero. Another rule, analogous to D&O’s Rule II, is the Widrow and Hoff (1960) and Johannet et al. (1992), an iterative rule based on gradient descent for obtaining the orthogonal projection matrix. The application of non-uniform increments in the weights, however, makes the hardware implementation of these rules more difficult.

Inspired by such iterative prescriptions, other works have been published describing methods for satisfying (5). Krauth and Mezard (1987) proposed the weakest-pattern first-update strategy, considering that the network should be trained focusing directly on the pattern with the worst alignment, which has the minimum value of $\sum_{i}^{N}h_{i}^{k}\,\xi_{i}^{k}$. Meanwhile, Gardner (1988) reported an identical implementation of the above-described Rule I, but this time resorting to alignment normalization. This normalization was achieved by using the root sum squared of the weights (that is to say, each weight matrix row was scaled using its norm). Both rules applied Hebbian weight updates.

In Tanaka et al. (2020), D&O’s Rule I was applied to the training of networks with specific interconnection architectures by forcing missing interconnections to have assigned weights equal to zero. The authors also explored how to derive networks minimizing the number of connections.

The problem is that, in general, these previous approaches lead to asymmetric weight matrices, containing widely dispersed floating-point (FP) values. Since the aim in our study was to obtain synaptic weight values that are physically implemented in the ONN, such solutions were of no interest. The proposed ONN-compatible learning rule is described in the section “2.2. Proposed learning rule: iterative random partial update symmetric hebbian (IRPUSH).”

### 2.2 Proposed learning rule: Iterative Random Partial Update Symmetric Hebbian (IRPUSH)

**FIGURE 2:**
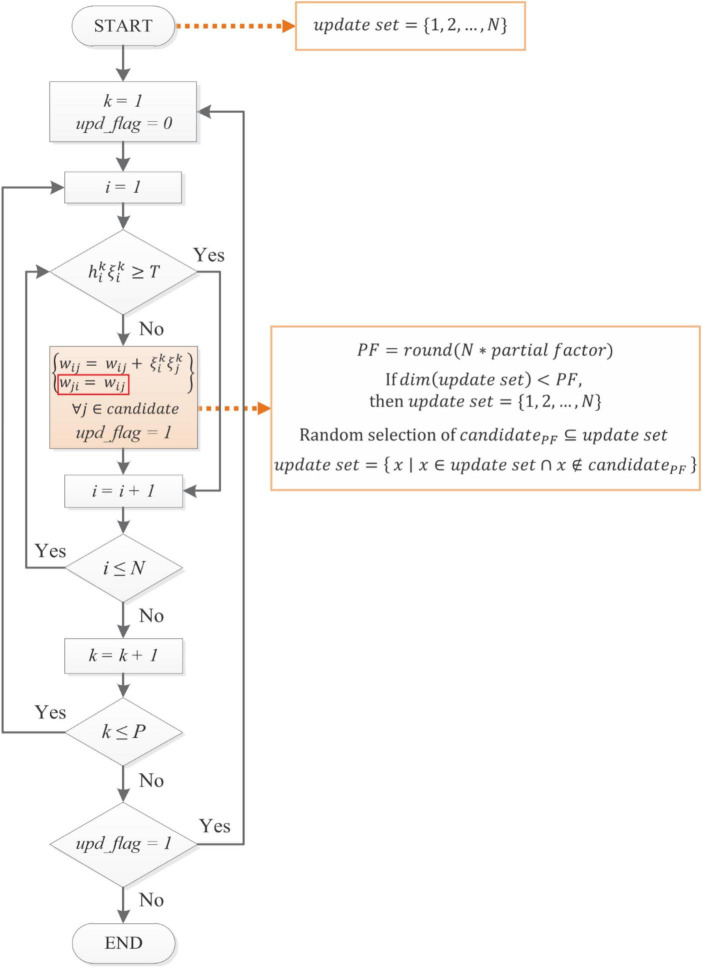
Proposed IRPUSH learning rule. It is based on Diederich and Opper (1987) but it enforces symmetry in the weight matrix and adds the partiality mechanism in the update step. Similarly, the synaptic weights of the evaluated neuron are partially adjusted whenever the corresponding *NP* embedding condition is not met. The number of modified weights (*candidate*_PF_) is determined by the partial factor (*PF*). The *candidate* weights are selected randomly from a set of indexes (*update set*).

Our proposed rule is based on D&O’s Rule I, described in the section “2.1. Hopfield neural network and learning rules,” and henceforth referred to as the Iterative Hebbian (IH) rule. Figure 2 shows the flow chart of the novel learning algorithm: Iterative Random Partial Update Symmetric Hebbian (IRPUSH). It consists of two nested loops, which are repeated until the algorithm converges to a solution that satisfies condition (5) (*upd_flag* = 0) or until a limit number of iterations is reached. The external loop is for patterns and the inner loop for neurons. This is exactly the same as in the IH rule. The key difference is the weight updating procedure.

First, in order to force symmetry in D&O’s algorithm, it is imposed that the new algorithm must have *w*_*ij*_ = *w*_*ji*_. In the IH rule, weight updates caused by neuron *i* not satisfying (5) have no impact on the compliance or non-compliance status of the remaining neurons because only *w*_*ij*_∀*j* ∈ 1,…,*N*/*j*≠*i* are modified. Due to the symmetry constraint, however, modification of *w*_*ij*_ implies modification of *w*_*ji*_, and so other neurons are also affected. From a different point of view, IH does not modify weights *w*_*ji*_ if neuron *j* satisfies (5) (for a given pattern), but this is not the case when symmetry is forced. This could negatively impact the performance of the learning rule since some neurons which already satisfied (5) might not fulfill it after the weight update forced by the symmetry constraint. In order to somehow counteract this effect, we propose that only a randomly selected fraction of the weights be updated at each step. This is motivated by Tanaka et al. (2020) which it states that a reduced number of connections while applying the iterative learning leads to a redistribution of the lost information to the remnant connections. We believe this can contribute to the resulting weight matrix being more suitable for the quantization.

The partial factor (*PF*) determines the number of connections that are randomly selected from the index *update set*. After the update step, the chosen indexes (*candidate*_*PF*_) are removed from the *update* set. All the indexes are restored back in the *update set* once it gets a lower number than the *candidate* length, imposed by the partial factor. The algorithm terminates when the *NP* inequalities are satisfied or a maximum number of training iterations is reached.

It is interesting to point out that the obtained matrix solution is always the same, in case of null partiality during the update step. With the partiality approach, a random fraction of the weights associated to the current evaluated neuron is modified with Hebbian learning. The randomness in this procedure allows to explore further different solutions in the weights assignment that totally complies with the embedding conditions of the training set when the algorithm converges.

### 2.3 Pattern recognition using associative memory

One of the most common AM tasks is pattern recognition. A network trained as an AM can recall stored patterns even when the network state is initialized with corrupted versions of them. A pattern is successfully retrieved when the closest one is retrieved, determined by the Hamming distance metric (HD). The Hamming distance metric (HD) measures the number of differing elements between two equal-length vectors, representing the minimum number of substitutions needed to transform one vector into the other. It can be defined as:


(8) $$H\,D\,\left(\xi^{\mu },\xi^{\rho }\right)=\frac{N-\left(\xi^{\mu }\cdot \xi^{\rho }\right)}{2}$$

The algorithmic model used follows a synchronous update scheme during a specific number of iterations. Depending on the initial input state, the output state may be: (a) the desired retrieved pattern, (b) an incorrect but stored pattern, (c) a spurious state pattern (a stable but undesired state), or (d) a limit cycle between two states.

The inferred steady state is considered correct when the HD is zero with the expected pattern. This criterion could be relaxed by allowing certain bit errors which could be tolerated without problems in some applications. In this study, however, success cases were considered exclusively using zero HD.

**FIGURE 3:**
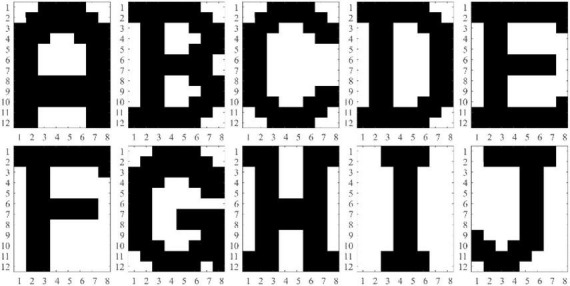
Training set with 12 × 8 patterns representing A–J characters.

To evaluate the performance of the HNN trained with IRPUSH in the pattern recognition task, a set of *P* = 10 correlated patterns of *N* = 96 (12 × 8) pixels representing characters A through J was chosen (see Figure 3). Table 1 shows the Hamming distances between the patterns from the training set, indicating their degree of correlation. Note that there are small HD values between some pairs of patterns (B-D, E-F, and C-G, marked in red), which make them harder to store in the AM.

**TABLE 1:** Hamming distances –12 × 8 characters.

	A	B	C	D	E	F	G	H	I	J
A		44	36	36	56	57	24	46	68	56
B	44		34	10	20	27	32	24	58	50
C	36	34		28	42	45	16	54	48	46
D	36	10	28		26	33	30	34	60	56
E	56	20	42	26		13	46	24	50	48
F	57	27	45	33	13		53	27	49	43
G	24	32	16	30	46	53		48	56	50
H	46	24	54	34	24	27	48		66	52
I	68	58	48	60	50	49	56	66		28
J	56	50	46	56	48	43	50	52	28	

The capacity and noise robustness of different trained 96-neuron networks were evaluated using several of the learning rule algorithms previously mentioned. Capacity was evaluated by training networks with an increasing number of patterns and moving in alphabetical order from character A to character J. Each network therefore had to show stability upon presentation of their stored patterns as the initial state and, likewise, upon presentation of their corresponding inverted versions, which also became fixed points or attractors in the network.

**FIGURE 4:**
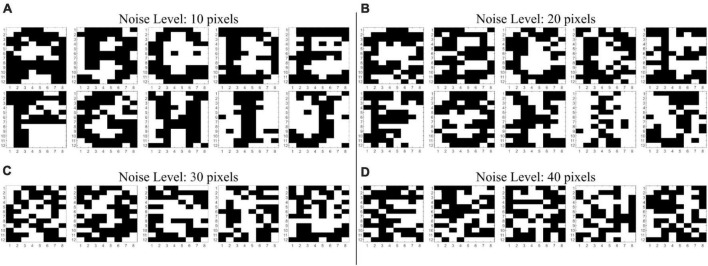
Noisy A–J patterns from the noise test set with **(A)** 10, **(B)** 20, **(C)** 30, and **(D)** 40 corrupted pixels. Beyond a noise level of 30 pixels, patterns are totally indistinguishable to the human eye.

The noise robustness analysis used a test set of 8,000 patterns. It was generated by randomly adding an increasing number of noisy pixels (that is, pixels with flipped value) to the 10 training patterns and their inverted versions. More specifically, 10 test patterns were generated for each training pattern and each noise level, which it is swept from “1 pixel to 40 flipped pixels.” There are 10 test patterns for each of the 10 training patterns and their inverted version, and these test patterns are generated at varying noise levels from 1 pixel to 40 flipped pixels. The total number of test patterns is 10 (test patterns per training pattern) × 20 (training patterns plus their inverted version) × 40 (different noise levels) = 8,000 test patterns. Note that this was also a difficult test set because of the high number of noisy pixels (40 out of 96) considered. Examples of test patterns with different numbers of noisy pixels are shown in Figure 4. Accuracy was measured in terms of how many test patterns returned a steady, successful inference of the nearest training pattern as indicated by the HD.

## 3 Results

### 3.1 Capacity evaluation

Different 96-neuron networks trained with our IRPUSH algorithm and using different values for parameter *T* were evaluated for storing the proposed set of 10 characters. The partial factor, which corresponds to the number of weights that are modified when a neuron is updated, was constrained to 100%: i.e., all 95 weights associated with one neuron being changed. Table 2 summarizes the capacity results obtained in this analysis and assesses the use of weights with reduced precision.

**TABLE 2:** Capacity with different *T*-values and weight precision.

*T*	IRPUSH, capacity (#)				IH, capacity (#)			
	FP	5-bit	4-bit	3-bit	FP	5-bit	4-bit	3-bit
0	**10**	**10**	9	6	**10**	8	7	0
10	**10**	**10**	**10**	5	**10**	**10**	9	7
50	**10**	**10**	**10**	8	**10**	**10**	**10**	8
110	**10**	**10**	**10**	9	**10**	**10**	**10**	9
150	**10**	**10**	**10**	**10**	**10**	**10**	**10**	9
200	**10**	**10**	**10**	**10**	**10**	**10**	**10**	9
400	**10**	**10**	**10**	**10**	**10**	**10**	**10**	8

The applied quantization method is based on: (1) normalization with respect to the maximum absolute weight value to obtain |*w*_*ij*_| ≤ 1. (2) Scaling by the largest absolute value which can be represented with the target number of bits (*nbit*) and (3) rounding. Note that the MSB bit is dedicated to the sign, while the remaining number of bits indicate the maximum integer value that can be represented: (2^*nbit*−1^−1).


(9) $$w_{i\,j}^{n\,b\,i\,t}=r\,o\,u\,n\,d\,\left(\frac{\left(2^{n\,b\,i\,t-1}-1\right)\,w_{i\,j}}{\max\left(\text{abs}\,\left(W\right)\right)}\right)$$

For purposes of comparison with a competitive candidate, the IH algorithm was also evaluated under similar conditions. Since update increments are not scaled by N, large *T*-values were chosen. Thus, case *T* = 1 in the original work corresponds to *T* = *N* in our implementation.

As can be seen in Table 2, every network retained the 10 patterns using FP weights. However, IRPUSH outperformed the IH capacity with 3-bit precision, being able to store the 10 patterns for largest values of *T*. IRPUSH also found solutions at lowest values of *T* with 5-bit and 4-bit precision.

A larger example with *N* = 1024 and *P* = 26 is evaluated in the Supplementary material.

### 3.2 Noise robustness results

As described in section “2.3. Pattern recognition using associative memory,” the benchmark for the noise robustness experiment consisted of 8,000 test patterns that were generated by applying increasing noise levels from 1 to 40 flipped pixels from the original training set. Each noise level had 200 associated test patterns.

**FIGURE 5:**
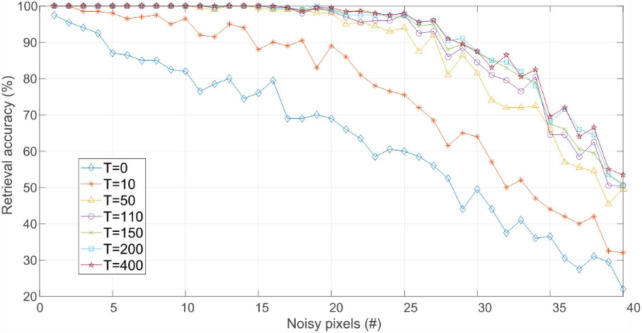
Retrieval accuracy vs. noise level for networks trained with the IRPUSH algorithm and different values of *T*.

Noise robustness was evaluated for those weight matrixes that store the 10 training patterns (shaded cells in Table 2). As previously described, the retrieval accuracy for this experiment was measured as the percentage of test patterns which HNN evaluation was correctly inferred. That is, the percentage of cases where the closest training pattern, indicated by the minimum HD, is retrieved Figure 5 shows the retrieval accuracy of networks trained with IRPUSH vs. noise level. It is evident that increasing the value of *T* produced larger basins of attraction, pushing forward the number of noisy pixels that the network could tolerate before the number of erroneous inferences began to rise. However, the benefits of increasing *T* were quickly reduced for *T* > 50, as intuitively observed in Figure 5.

The accuracy results obtained with IRPUSH and IH and different *T*-values are reported in Table 3, together with their performance with limited precision. The advantages of IRPUSH were greatly reduced as *T* increased, displaying very similar performance as IH. IRPUSH accuracy loss at 4-bit precision remained under 3% with respect to FP, while accuracy in 3-bit cases was degraded by around 20%. On the other hand, the 4-bit IH presented accuracy degradation of up to 6% with respect to FP.

**TABLE 3:** Retrieval accuracy with different *T* values and weight precision.

*T*	IRPUSH, accuracy (%)				IH, accuracy (%)			
	FP	5-bit	4-bit	3-bit	FP	5-bit	4-bit	3-bit
0	63.4	57.7	–	–	37.1	–	–	–
10	76.2	70.5	77.1	–	57.8	53.4	–	–
50	87.8	86.9	85.6	–	83.6	83.0	80.5	–
110	89.7	89.6	87.9	–	89.5	87.7	86.7	–
150	90.6	90.2	88.3	67.0	90.1	89.0	84.6	–
200	91.2	90.4	89.2	71.3	91.0	90.9	86.3	–
400	91.5	90.3	89.5	72.0	91.6	91.1	89.3	–

Keeping in mind that IRPUSH was used with a partial factor equal to 100%, the only difference with respect to IH was that IRPUSH constrained the weight matrix to be symmetric. The results show that imposing symmetry did not penalize the accuracy obtained obtained, as indicated in section “2.2. Proposed learning rule: iterative random partial update symmetric hebbian (IRPUSH),” where the enforcement of symmetry was observed to potentially hinder the performance of IRPUSH. In fact, our approach obtained better results for lower *T*-values. The better accuracy results described above were probably a side effect of the larger number of update operations resulting from having imposed symmetry. Unlike the original asymmetric update in IH, *w*_*ij*_ (and *w*_*ji*_) could both be modified when processing neuron *i* or neuron *j*. Of even greater interest, however, was the success of our approach in storing the ten patterns with 3-bit weights, as pointed out in the capacity evaluation, for *T* ≥ 150.

The next step was to evaluate IRPUSH for partial factors below 100% so that the proposed random partial update mechanism was actually applied.

### 3.3 Impact of random partial update mechanism

The networks were also evaluated by applying the philosophy of updating a reduced number of weights, each time an update step took place. They had a common *T*-value of 95 (corresponding to set Rule I’s threshold to unity). The partial factor was reduced from 100% to 1% in steps of 25%, plus the selected factors of 33% and 10%.

All the networks stored the training set perfectly. Moreover, the lower the partial factor, the larger the number of training iterations that was required to satisfy the embedding of the patterns.

**FIGURE 6:**
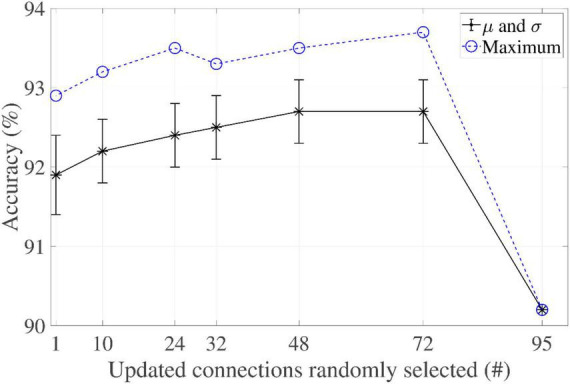
Partial factor evaluation of IRPUSH (*T* = 95).

It is interesting to note that the algorithm returns a different weight matrix solution each time it is run, because the connections to be updated are selected randomly. A battery of 100 experiments for each case was therefore carried out. Figure 6 reports their respective maximum, average, and standard deviation values. Note that the case in which all neurons were updated (100%), an identical solution was therefore returned every time. It was found that reducing the number of updated connections resulted in an increase in accuracy with regard to IRPUSH with a partial factor of 100%.

The impact of *T* was also explored for two particular cases: partial factors of 33% and 25%. Table 4 shows the accuracy results for different *T*-values and weight precisions. It is worth noting that IRPUSH with the random partial update mechanism was quite satisfying: not only in the number of cases storing the 10 patterns, but also in improving accuracy when comparing with Table 3, even with 3-bit precision. With the 33% (25%) partial factor at *T* = 150 (*T* = 110), a best record value of 90.9% (87.5%) was achieved for this precision. This best value for IRPUSH without the partial update mechanism and 3-bit precision is only 72%. Solutions using 4-bit weights with a partial update factor of 33% experienced accuracy degradation of less than 1% with respect to FP.

**TABLE 4:** Accuracy of IRPUSH vs. *T* with partial update.

*T*	Accuracy, 33% factor (%)				Accuracy, 25% factor (%)			
	FP	5-bit	4-bit	3-bit	FP	5-bit	4-bit	3-bit
0	39.0	40.4	35.0	–	30.5	30.5	–	–
10	80.7	82.0	80.9	–	82.4	81.0	78.2	–
50	91.7	91.4	90.8	**84.4**	90.8	90.9	89.5	**84.0**
110	92.6	92.4	91.9	**90.2**	93.1	92.7	91.9	**87.5**
150	92.7	93.0	92.5	**90.9**	92.8	92.4	93.5	**75.0**
200	93.2	93.0	92.2	**83.3**	92.9	92.7	92.2	**84.6**
400	93.4	93.2	93.3	–	92.3	92.5	93.3	82.6

Comparing the results obtained for the most reduced numbers of bits with partial update (Table 4) and without (Table 3), it can be observed that much better results are achieved with partial update. For example, accuracy up to 90.9% with 33% of partial factor and three bits is obtained while the best results without partial update and that precision is only 72%. That is, the results support our hypothesis concerning the benefits of the partial update procedure.

**FIGURE 7:**
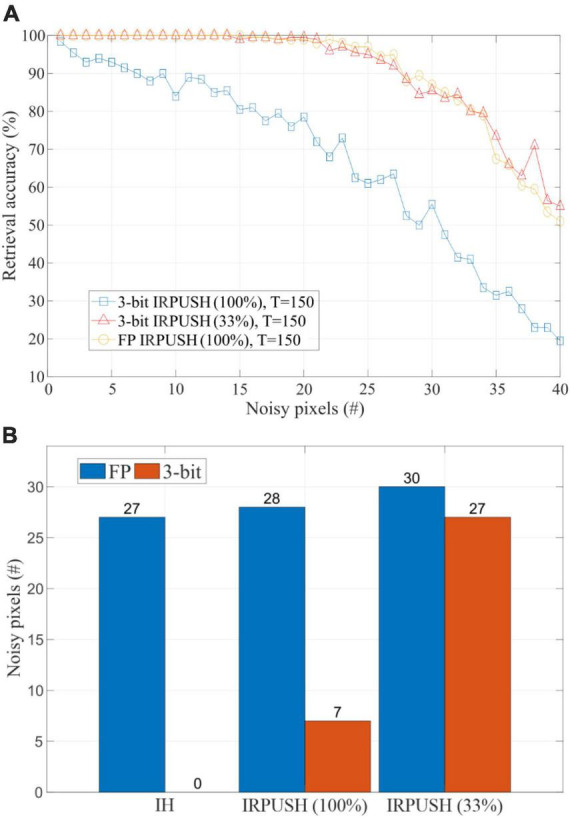
**(A)** Retrieval accuracy vs. noise level using IRPUSH with partial factors of 100% and 33% and *T* = 150. **(B)** Maximum noise level tolerated keeping retrieval accuracy above 95% using IH and IRPUSH with partial factors of 100% and 33%. Considered *T*-value was the one providing the best result for each case.

To further compare the inference robustness of the IRPUSH algorithm against noise, Figure 7A depicts the retrieval performance of IRPUSH with partial factors of 100% and 33% against different noise levels for the same *T*-value of 150. Similar results were obtained with FP and a partial factor of 100% and with 3-bit precision and a partial factor of 33%. Both outperform 3-bit precision with a partial factor of 100%. This clearly illustrates the advantage of the random partial update mechanism using limited precision and demonstrates its ability to avoid serious degradation of the retrieval capabilities under low weight precision, when compared with the FP precision case. Figure 7B compares the maximum number of noisy pixels for which the evaluated networks achieved an accuracy greater than 95%. It shows the best result obtained among different *T-*values in each case. The partial update strategy for 3-bit weights had a major impact, raising this figure of merit from 7 to 27.

### 3.4 Comparison of learning rules

Other learning rules that managed to store the complete set of training patterns have been studied in the noise robustness experiment. One-shot Hebbian and Storkey learning rules, for example, were only able to store up to 3 and 4 patterns, respectively. Both were therefore discarded, along with other one-shot rules. In contrast, the few-shot training approach with the Storkey rule successfully embedded the 10 training patterns as attractors, reinforcing the assumption that repetitive learning enhances memory capabilities. The pseudoinverse rule also produced perfect results in terms of capacity. In the same manner, asymmetric solutions based on the Krauth and Mezard (1987) algorithm with Gardner normalization (Gardner, 1988) (GKM) and on an optimization solver with Descent Exponential Barrier (DEB) as the target objective function were also evaluated. All of them could store the 10-pattern set with FP, 5-bit, and 4-bit precision. The only rule capable of storing 10 patterns with 3-bit precision, however, was the one proposed in this work.

**FIGURE 8:**
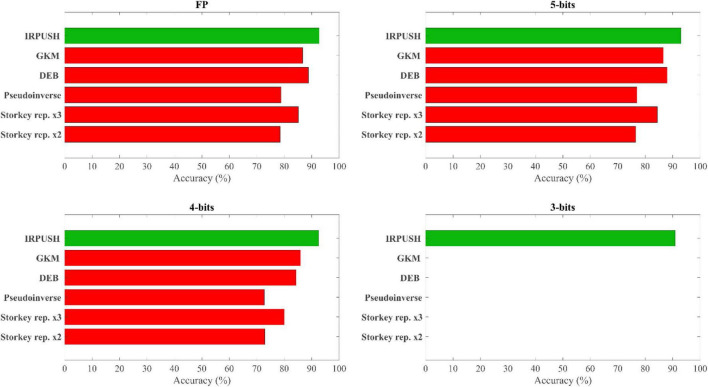
Retrieval accuracy comparison with other learning rules.

Figure 8 summarizes the accuracy results obtained with different weight precisions. With FP weights, the network using Storkey weights with two repetitions of the training set achieved almost 80% accuracy, similar to that obtained with the pseudoinverse solution. Both of these rules, however, were outperformed by the Storkey solution with three repetitions, which achieved an accuracy of up to 85%. As expected, accuracy decreased when these rules were applied with 5-bit and 4-bit weight precisions, although it is worth noting that the loss of accuracy was less than 6% when 4-bit precision was used instead of FP precision with the three mentioned solutions. For FP and 5-bit precision, the rule based on the optimization solver, DEB, proved to be more accurate than the other rules from literature included in the study, while for 4-bit precision it was narrowly surpassed only by the iterative solution based on GKM. Both of these solutions showed a reduced loss of accuracy in comparison with their respective FP results: the 4-bit DEB experienced a degradation of 4.5% and the 4-bit GKM one of 0.8%. Clearly, the best results (green color in Figure 8) for each precision were obtained with the solution proposed in this paper: IRPUSH.

## 4 Discussion

Despite the affinity of single-layer ONNs with HNNs, many learning rules reported in the extensive HNN learning literature for associative memory operation are not compatible with the constraints imposed by analog ONN implementations. This study therefore proposed and evaluated a novel algorithm, IRPUSH, suitable for training such analog ONNs. The new algorithm was developed on the basis of an iterative approach to solving the set of inequalities that define the pattern embedding condition, and works by imposing the required symmetry condition on the weight matrix. The distribution of information along synapses was improved by adopting a random partial update strategy. The proposed algorithm was evaluated together with well-known HNN learning rules on a pattern recognition task involving a 12 × 8 “A-J” character set. Storing a set of this type is much harder than using non-correlated random patterns. As previously mentioned, the implementation of analog ONN synapses is electrically constrained, and the evaluated solutions were therefore assessed under reduced precision. Of all the rules analyzed, IRPUSH was the only one capable of providing a solution with 3-bit weight precision. Furthermore, this solution has a remarkably low accuracy loss of 2% to 5% with respect to FP. The best accuracy obtained with 3-bit precision was 91% for IRPUSH with a 33% update factor. This retrieval accuracy is 8% higher than that of a 4-bit solution produced by the original iterative approach. IRPUSH also has attractive features for online learning, such as fixed weight increments.

## Data availability statement

The raw data supporting the conclusions of this article will be made available by the authors, without undue reservation.

## Author contributions

MJ: Conceptualization, Methodology, Software, Validation, Formal analysis, Writing – original draft, Writing – review and editing, Visualization. MA: Conceptualization, Formal analysis, Writing – review and editing, Supervision. BL-B: Writing – review and editing, Supervision, Funding acquisition. JN: Writing – review and editing, Visualization, Supervision.
