# Supplementary material for: Learning algorithms for oscillatory neural networks as associative memory for pattern recognition
Source: Front Neurosci. 2023 Nov 29;17:1257611. doi: 10.3389/fnins.2023.1257611 (PMC10716297; doi:10.3389/fnins.2023.1257611)
Supplement: Supplementary file 1 [file Data_Sheet_1.pdf]

**Learning Algorithms for Oscillatory Neural Networks as Associative Memory for Pattern Recognition**

**Manuel Jiménez, María J. Avedillo, Bernabé Linares-Barranco, Juan Núñez**

# Supplementary Material

## 1 Pattern embedding condition

Considering the activation function of the Hopfield Neural Network for one neuron´s state:

$$s_i = sign(h_i) = sign\left(\sum_{j=1}^{N} w_{ij} s_j\right)$$

For a given training pattern, $\xi^k \epsilon \{-1, +1\}^N$, to be a fixed point of the network (that is, to be stored), it is required to accomplish:

$$\xi_i^k = sign\left(h_i^k\right) = sign\left(\sum_{j=1}^{N} w_{ij} \xi_j^k\right) \; \forall \; i \in 1, \dots, N$$

That can be understood as the sign alignment of $h_i^k$ and $\xi_i^k$.

Thus, the embedding conditions of a set of *P* training patterns can be written as the system of *NP* inequalitites:

$$h_i^k \xi_i^k > 0 \quad \begin{array}{l} \forall \; i \in 1, \dots, N \\ \forall \; k \in 1, \dots, P \end{array}$$

Additionally, it is easy to prove that the inverted version of a stored pattern is also a fixed point of the network. Given a stored pattern, $\xi^k$, its inverted can be denoted as $-\xi^k$ and the hidden potential is:

$$h_i^k = \sum_{j=1}^{N} w_{ij}\left(-\xi_j^k\right) = -\sum_{j=1}^{N} w_{ij} \xi_j^k$$

The evaluation of the inverted pattern returns the same potential magnitude as with the original pattern but with opposite sign, then the embedding conditions are be accomplished.

$$\sum_{j=1}^{N} w_{ij}\left(-\xi_j^k\right)\left(-\xi_{ji}^k\right) > 0$$

## 2 D&O´s Rule I and IRPUSH

Two simple iterative methods for solving the *NP* inequations, which comprise the pattern-embedding problem, were presented in (Diederich, 1987). The first is called D&O´s Rule I and its corresponding diagram flow is depicted in Figure S1A.

$$\sum_{j=1}^{N} w_{ij}\xi_j^k \, \xi_i^k = h_i^k \xi_i^k > T \quad \begin{array}{l} \forall\, i \in 1, \dots, N \\ \forall\, k \in 1, \dots, P \end{array}$$

Weights ($w_{ij}$) are obtained by solving iteratively this system of *NP* constraints, when the *i*-th neuron potential ($h_i^k$), under the presentation of the *k*-th training pattern, is sign-aligned with the desired value, $\xi_i^k$. Successful learning of the training patterns occurs when all *N* neurons satisfy the conditions for all *P* patterns. The threshold $T > 0$ is employed to increase the basins of attraction associated to the stored patterns. It ensures the potential, $h_i^k$, to be greater than the minimum required to make them fixed points of the network, contributing to enhance their attractor capability.

As shown in the diagram flow, when the *i*-th neuron does not fulfill the condition, its synaptic weights are modified following the Hebbian rule. This procedure is sequentially iterated over every neuron and training pattern, leading to an asymmetric weight matrix solution. This learning rule can be summarized using the Heaviside function, $\theta$, as follows:

$$\Delta w_{ij} = \frac{\xi_j^k \xi_i^k}{N-1}\theta(T - h_i^k \xi_i^k) \quad \begin{array}{l} \forall\, i \in 1, \dots, N \\ \forall\, k \in 1, \dots, P \end{array}$$

The proposed learning rule, IRPUSH, is inspired by D&O´s Rule I and is depicted in Figure S1B. It also evaluates iteratively the *NP* embedding conditions to determine when a neuron requires a modification of its synaptic weights. Once it occurs, a selection of the neuron´s weights is adjusted applying the Hebbian rule. The indexes of the selected weights (*candidate*) are randomly chosen from a set of indexes (*update set*) and the number of selected indexes is determined by the partial factor. The symmetry in the matrix is imposed each time a weight is updated, so the respective weight assigned to the other neuron has the same value ($w_{ji} = w_{ij}$). Also, IRPUSH algorithm sets a limit number of iterations to terminate in case of no convergence, which is not represented in the diagram.



This is a provisional file, not the final typeset article

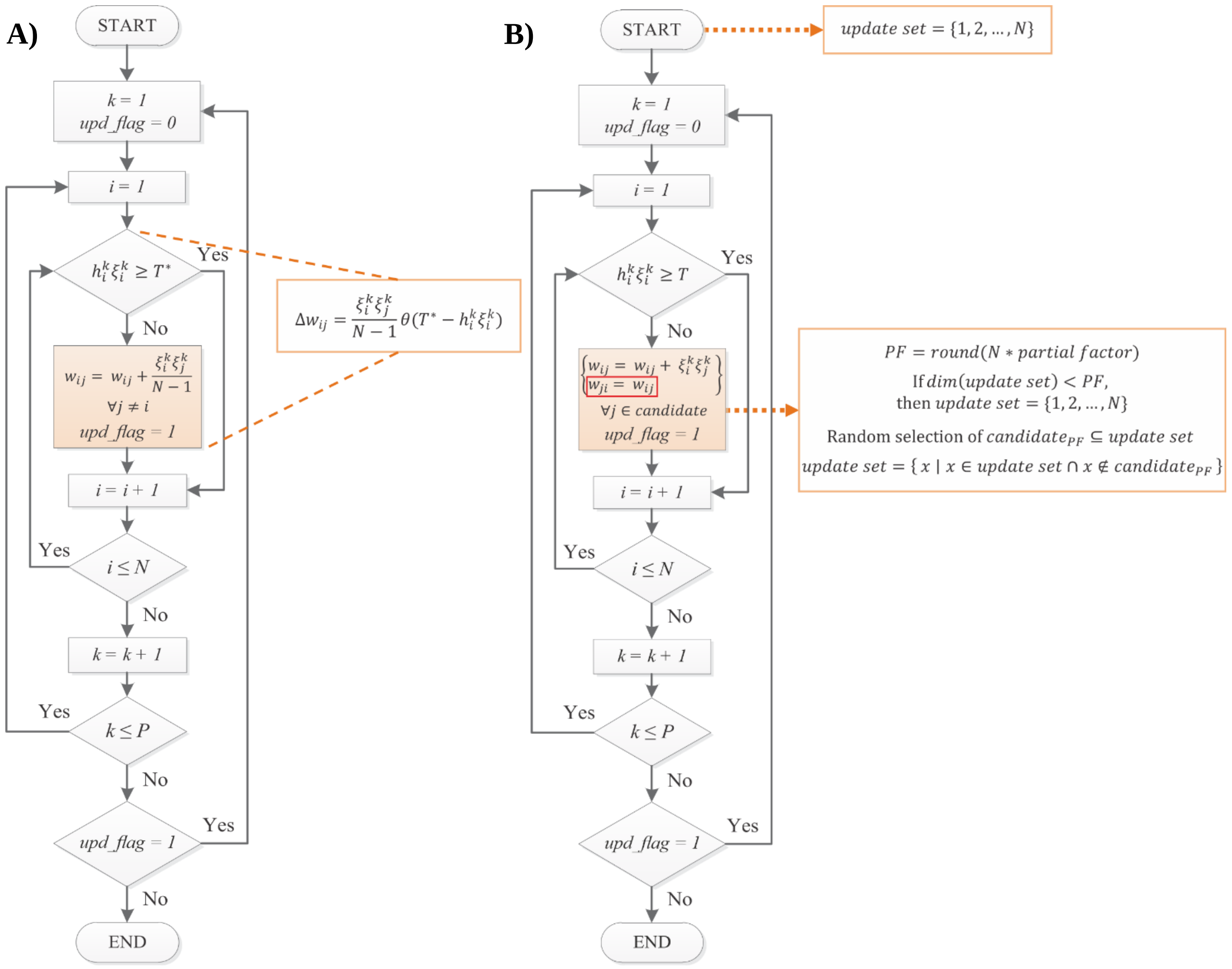


**Figure S1. A)** First iterative method proposed in (Diederich, 1987). It conducts a sequential modification of the synapses with the Hebbian learning rule when the evaluated neuron fails the inequality that ensures the retaining of the desired pattern information to store. The update rule is summarized in the inserted box where *θ(x)* stands for the Heaviside function. Successful learning of the training patterns occurs when all *N* neurons satisfy the conditions for all *P* patterns. The parameter $T > 0$ is employed to increase the basin of attraction. Notation $T^*$ is used to distinguish from the parameter *T* used in the IRPUSH algorithm, where the Hebb increment is not scaled with the number of neurons, *N*. **B)** Proposed IRPUSH learning rule. It is based on (Diederich, 1987) but it enforces symmetry in the weight matrix and adds the partiality mechanism in the update step. Similarly, the synaptic weights of the evaluated neuron are partially adjusted whenever the corresponding *NP* embedding condition is not met. The number of modified weights (*candidate*) is determined by the partial factor (*PF*). The *candidate* weights are selected randomly from a set of indexes (*update set*).

## 3 32x32 Characters Training Set: A Capacity Case Study

To further explore the capabilities of the IRPUSH algorithm to store patterns a larger case study has been evaluated. A training set of *P*=26 patterns (Figure S2) with 32x32 (*N*=1024) pixels have been used.

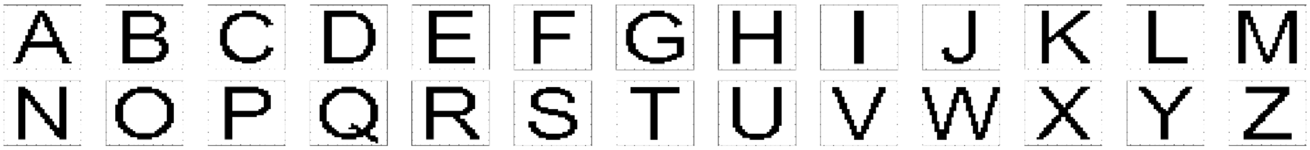


**Figure S2.** Characters A-Z, binary patterns with dimension 32x32 used as training patterns.

Along with IRPUSH and IH, different learning rules were evaluated as Hebb, Storkey, Pseudoinverse, or based on optimization solver of the gradient descent. Many of them were not able to store the 26 patterns, even using weights with Full-Precision (FP). Also, the partial mechanism allowed by IRPUSH is explored. Table S1 depicts the minimum weight precision required for those of them which succeeded in storing the 26 characters.

Storkey approach with several repetitions is able to store the 26 patterns when the training set is presented 8 times, but is not able to be discretized lower than 9 bits. The Pseudoinverse and the optimization solver using L2 distance metric success with 6-bit weight precision. IH and IRPUSH without applying partiality is able to retain the 26 patterns for any value of $T$ with FP and are able to work with different precision depending on the value of $T$. For example, weight matrix obtained with IH and $T \geq 110$ can be quantized to 7 bits, while IRPUSH also achieves that precision using $T \geq 50$. Furthermore, IRPUSH is successful with 6-bit employing $T \geq 400$.

Descent Exponential Barrier (DEB) and IRPUSH with the random partial mechanism are able to reduce the required bit precision up to 5 bits. It´s worth to highlight that the computation time of IRPUSH is few seconds while the optimization solver algorithm, DEB, took almost one hour running on the Google Collaboratory environment using a Python-Jupyter Notebook.

**Table 1.**
**Comparison of minimum number of bits required to store the 26 patterns.**

| Rule Description | # bits required |
|---|---|
| **Storkey rep. x8** | 9 |
| **Pseudoinverse** | 6 |
| **Descent L2** | 6 |
| **DEB** | 5 |
| **IH ($T \geq 110$)** | 7 |
| **IRPUSH ($T \geq 50$, 100% partial factor)** | 7 |
| **IRPUSH ($T \geq 400$, 100% partial factor)** | 6 |
| **IRPUSH ($T \geq 10$, 33% factor)** | 5 |
| **IRPUSH ($T \geq 0$, 25% factor)** | 5 |


This is a provisional file, not the final typeset article

Table S2 compares IRPUSH without partial update and IH for different T values. Two different parameters are shown. The minimum weight precision able to store the 26 characters and the number of learning iterations required by each algorithm.

**Table 2.**
**Minimum number of bits and learning iterations with different *T* values for IH and IRPUSH without partial update.**

| | # bits required | | # iterations | |
|---|---|---|---|---|
| ***T*** | **IH** | **IRPUSH (100%)** | **IH** | **IRPUSH (100%)** |
| **0** | (>12) | 10 | 9,844 | 8,093 |
| **10** | 10 | 8 | 10,137 | 8,380 |
| **50** | 8 | 7 | 11,186 | 9,123 |
| **110** | 7 | 7 | 13,025 | 10,513 |
| **150** | 7 | 7 | 14,121 | 11,356 |
| **200** | 7 | 7 | 15,188 | 12,594 |
| **400** | 7 | 6 | 20,856 | 17,181 |

It can be observed as in the simpler example in the main text of the paper that increasing the threshold value used to derive the weight matrix allows reducing the weight precision. Also, the superiority of IRPUSH is clear. A solution with just 6 bits was found for IRPUSH but not for IH in our experiment. IH requires a larger number of iterations than IRPUSH.

Table S3 compares IRPUSH with no partial update to IRPUSH with 33% partial factor. Advantages of IRPUSH (33%) in terms of number of bits required is clearly observed. It uses a higher number of iterations than IH for the large *T* values when the random update is applied. This is due to a reduced increment of the neuron potential, $h_i^k$, per each update since a smaller number of weights are modified, therefore requiring more iterations to reach the threshold *T*.

**Table 3.**
**Minimum number of bits and learning iterations with different *T* values for IRPUSH using a partial factor of 33% and 100% (no partial update).**

| | # bits required | | # iterations | |
|---|---|---|---|---|
| ***T*** | **IRPUSH (33%)** | **IRPUSH (100%)** | **IRPUSH (33%)** | **IRPUSH (100%)** |
| **0** | (>6) | 10 | 7,007 | 8,093 |
| **10** | 5 | 8 | 7,467 | 8,380 |
| **50** | 5 | 7 | 9,293 | 9,123 |
| **110** | 5 | 7 | 12,171 | 10,513 |
| **200** | 5 | 7 | 16,450 | 12,594 |
| **400** | 5 | 6 | 26,107 | 17,181 |
